# Supplementary material for: Engineering Metal–Organic Frameworks (MOFs) for Controlled Delivery of Physiological Gaseous Transmitters
Source: Nanomaterials (Basel). 2020 Jun 8;10(6):1134. doi: 10.3390/nano10061134 (PMC7353332; doi:10.3390/nano10061134)
Supplement: Supplementary file 1 [file nanomaterials-10-01134-s001.pdf]

Table S1. Summary of gaseous transmitter-delivering MOFs involved in this review.

Abbreviation	Chemical Compositions	Stability	NO-Releasing Profiles	Biomedical Applications	Toxicity	In vitro/vivo Studies	Ref
**MIP-177**	$Ti(iPrO)_4$; H_4mdip	< 9% degradation within 72 h in biological media	1.7 μmol/mg	Wound healing	No cytotoxicity at 90 μg/mL	HeLa cells	[1]
**Ni-MOF**<br>**Co-MOF**	$NiCl_2{\cdot}6H_2O$; vitamin B_3/ $CoCl_2{\cdot}6H_2O$; vitamin B_3	Stable in water	2.6 μmol/mg<br>2.0 μmol/mg	Wound healing	Low toxicity at concentrations below 180 μg/mL	HeLa cells; HEKn cells	[2]
**CPO-27**	Mg^{2+}; Zn^{2+}; Ni^{2+}; $Na_2(dhtp){\cdot}2H_2O$			Vasodilation		Artery relaxation tests	[3]
**NO@HKUST-1**	Cu^{2+}; H_3btc;	Stable in a dry environment	1.74 nmol/L/h for more than 14 d	Diabetic wound healing; angiogenesis	High biocompatibility	C57BL/6J mice;	[4]
**IRMOF-3–NONO**<br>**UMCM-1–NONO**	IRMOF-3<br>UMCM-1-NH_2	Unstable in aqueous media	0.51 ± 0.11 mmol/g<br>0.10 ± 0.01 mmol/g				[5]
**Cu-TDPAT**	Cu^{2+}; H_6TDPAT	Stable for months under dry conditions at RT	175 μmol/g Cu-TDPAT after 7 days at 85% relative humidity and RT	Wound healing and organ preservation			[6]
**NOF-1/**<br>**NOF-2**	Zn^{2+}; 2nIm<br>Zn^{2+}; mnIm		3.4 μmol/mg<br>2.9 μmol/mg	Regulation of intracellular Ca^{2+} ions concentration	NO notable cytotoxicity	HEK293<br>HEK293-TRPC5	[7]
**NOF-11/**<br>**NOF-12**	$TiO(iPr)_4$; MeNH-bdc<br>$AlCl_3{\cdot}6H_2O$; MeNH-bdc	NOF-11 is highly stable under water and physiological conditions	1.60 mmol/g<br>2.78 mmol/g	Controlled NO release			[8]
**NMOF-SNO**	Zr^{4+}; Mn-TCPP		Not quantified	Magnetic resonance imaging; photothermal therapy	More than 90% cells viability at concentrations below 200 μg/mL	MCF-7; BALB/c mice	[9]
**Cu-BTTri/PVA**	Cu-BTTri; PVA		maximum NO flux: 0.20 ± 0.02 nmol/cm^2/min (10 wt.%)	NO release from GSNO at physiological temperature and pH			[10]
**Cu-SURMOFs**	$Cu(OAc)_2$; BTC	Increased stability with increased deposition cycles in PBS (pH 7.4)	The NO flux increased with the numbers of deposition cycles	Good catalytic durability; prevented thrombosis; reduced neointimal hyperplasia	Cell apoptosis	EC cell; SMC; RAW264.7; New Zealand white rabbits	[11]
**MIL-88B-Fe/**<br>**NH_2-MIL 88B-Fe**	$FeCl_3{\cdot}6H_2O$; H_2BDC<br>$FeCl_3{\cdot}6H_2O$; NH_2-BDC	Decomposition requires up to 4 h in PBS (pH 7.4)	0.36 μmol CO/mg;<br>0.69 μmol CO/mg		Good biocompatibility	Myoglobin assay	[12]

**CORF-1**	$MnBr(bpy)(CO)_3$; UiO-67-bpy	Decomposition in PBS	4.65 mmol CO/g	Anti-inflammation	Almost no cytotoxicity	HeLa cells	[13]
**Ni-CPO/ Zn-CPO**	Ni^{2+}; H_4dhtp Zn^{2+}; H_4dhtp	Moisture-sensitive	1.8 mmol H_2S/g and 0.5 mmol H_2S/g after 30 min in a moist atmosphere	Vasodilatory action	Ni-CPO is more toxic than Zn-CPO	Pig coronary arteries	[14]

References

1. Pinto, R.V.; Wang, S.; Tavares, S.R.; Pires, J.; Antunes, F.; Vimont, A.; Clet, G.; Daturi, M.; Maurin, G.; Serre, C.; et al. Tuning cellular biological functions through the controlled release of NO from a porous Ti-MOF. *Angew. Chem. Int. Ed.* **2020**, *59*, 5135–5143.
2. Pinto, R.V.; Antunes, F.; Pires, J.; Graça, V.; Brandão, P.; Pinto, M.L. Vitamin B3 metal–organic frameworks as potential delivery vehicles for therapeutic nitric oxide. *Acta Biomater.* **2017**, *51*, 66–74.
3. Cattaneo, D.; Warrender, S.J.; Duncan, M.J.; Kelsall, C.J.; Doherty, M.K.; Whitfield, P.D.; Megson, I.L.; Morris, R.E. Tuning the nitric oxide release from CPO-27 MOFs. *RSC Adv.* **2016**, *6*, 14059–14067.
4. Zhang, P.; Li, Y.; Tang, Y.; Shen, H.; Li, J.; Yi, Z.; Ke, Q.; Xu, H. Copper-based metal–organic framework as a controllable nitric oxide-releasing vehicle for enhanced diabetic wound healing. *ACS Appl. Mater. Interfaces* **2020**, *12*, 18319–18331.
5. Nguyen, J.; Tanabe, K.; Cohen, S. Postsynthetic diazeniumdiolate formation and NO release from MOFs. *Crystengcomm* **2010**, *12*, 2335–2338.
6. Lowe, A.; Chittajallu, P.; Gong, Q.; Li, J.; Balkus, K.J. Storage and delivery of nitric oxide via diazeniumdiolated metal organic framework. *Microporous Mesoporous Mater.* **2013**, *181*, 17–22.
7. Diring, S.; Wang, D.O.; Kim, C.; Kondo, M.; Chen, Y.; Kitagawa, S.; Kamei, K.I.; Furukawa, S. Localized cell stimulation by nitric oxide using a photoactive porous coordination polymer platform. *Nat. Commun.* **2013**, *4*, 2684.
8. Kim, C.; Diring, S.; Furukawa, S.; Kitagawa, S. Light-induced nitric oxide release from physiologically stable porous coordination polymers. *Dalton Trans.* **2015**, *44*, 15324–15333.
9. Zhang, H.; Tian, X.-T.; Shang, Y.; Li, Y.-H.; Yin, X.-B. Theranostic Mn-porphyrin metal–organic frameworks for magnetic resonance imaging-guided nitric oxide and photothermal synergistic therapy. *ACS Appl. Mater. Interfaces* **2018**, *10*, 28390–28398.
10. Neufeld, M.J.; Lutzke, A.; Jones, W.M.; Reynolds, M.M. Nitric oxide generation from endogenous substrates using metal–organic frameworks: Inclusion within Poly(vinyl alcohol) membranes to investigate reactivity and therapeutic potential. *ACS Appl. Mater. Interfaces* **2017**, *9*, 35628–35641.
11. Zhao, Q.; Fan, Y.; Zhang, Y.; Liu, J.; Li, W.; Weng, Y. Copper-based SURMOFs for nitric oxide generation: Hemocompatibility, vascular cell growth, and tissue response. *ACS Appl. Mater. Interfaces* **2019**, *11*, 7872–7883.
12. Ma, M.; Noei, H.; Mienert, B.; Niesel, J.; Bill, E.; Muhler, M.; Fischer, R.A.; Wang, Y.; Schatzschneider, U.; Metzler-Nolte, N. Iron metal–organic frameworks MIL-88B and NH2-MIL-88B for the loading and delivery of the gasotransmitter carbon monoxide. *Chem. Eur. J.* **2013**, *19*, 6785–6790.
13. Diring, S.; Carné-Sánchez, A.; Zhang, J.; Ikemura, S.; Kim, C.; Inaba, H.; Kitagawa, S.; Furukawa, S. Light responsive metal–organic frameworks as controllable CO-releasing cell culture substrates. *Chem. Sci.* **2017**, *8*, 2381–2386.
14. Allan, P.K.; Wheatley, P.S.; Aldous, D.; Mohideen, M.I.; Tang, C.; Hriljac, J.A.; Megson, I.L.; Chapman, K.W.; De Weireld, G.; Vaesen, S.; et al. Metal–organic frameworks for the storage and delivery of biologically active hydrogen sulfide. *Dalton Trans.* **2012**, *41*, 4060–4066.
